# Imitation learning for legged robot locomotion: a survey

**DOI:** 10.3389/frobt.2025.1678567

**Published:** 2025-12-01

**Authors:** Khojasteh Z. Mirza, Shubham Singh

**Affiliations:** 1 Friedman Brain Institute, Icahn School of Medicine at Mount Sinai, New York, NY, United States; 2 The University of Texas at Austin, Austin, TX, United States

**Keywords:** imitation learning, reinforcement learning, legged robotics, locomotion control, sim-to-real transfer

## Abstract

Imitation learning (IL) has fundamentally transformed the field of legged robot locomotion, removing the dependence on hand-engineered reward functions. Since 2019, this area of research has progressed rapidly, from simple motion-capture replication to the generation of sophisticated policies using diffusion models. This survey offers a comprehensive analysis of 35 pivotal research works, using a structured six-dimensional framework to investigate advancements using quadrupedal and humanoid platforms. The review also pinpoints significant challenges related to deployment and outlines new research directions. A key finding from the survey indicates that behavior cloning is utilized in almost half of the analyzed studies. Moreover, data generated through model-predictive control (MPC) now represents the most frequently used training data source for advanced imitation learning systems.

## 1 Introduction

Modern legged robotics has achieved remarkable milestones—robots now navigate rocky terrain, ascend staircases, and perform industrial door manipulation tasks. These achievements stem from three fundamental control paradigms: model-predictive control (MPC) for optimization-based approaches, reinforcement learning (RL) for reward-driven policy development, and imitation learning (IL), where robots acquire skills by directly replicating expert demonstrations.

Unlike reinforcement learning, which requires careful reward engineering, imitation learning offers compelling advantages: accelerated development cycles, reduced hyperparameter sensitivity, and natural scalability, when demonstration data are abundant. Gu et al. (2025) provided a broader coverage of humanoid control methods across planning and learning domains. Another recent survey specifically on imitation learning for general contact-rich tasks was given by Tsuji et al. (2025) who addressed a central challenge in robotics: *enabling robots to perform tasks involving continuous physical contact*, such as assembly, insertion, polishing, and manipulation of deformable objects. In that context, our paper focuses mostly on legged locomotion for humanoids, quadrupeds, and hybrid-form robots.

The imitation learning landscape has considerably diversified since 2019. What began as simple paradigm of learning from demonstrations has now branched into six distinct methodological families:

1. Behavioral cloning (BC): Direct supervised learning from state–action pairs
2. Adversarial motion priors (AMPs/GAIL): Discriminator-based realism enforcement
3. Diffusion models (DIFs): Probabilistic denoising for multimodal action generation
4. Decaying action priors (DAPs): Time-dependent teacher guidance mechanisms
5. MPC distillation (MPC): Physics-informed MPC knowledge transfer
6. Curricular hindsight reinforcement learning (CHRL): Imitation-seeded progressive learning
7. Mimic (MM): Physics-based motion imitation baseline in character animation

### 1.1 Research scope and contributions

This survey analyzes 35 key papers, most of them published between 2019 and mid-2025, focusing exclusively on pure imitation approaches without initial reward shaping. Our analysis provides the following:

1. Systematic classification: A multi-dimensional taxonomy enabling rapid literature filtering based on robot morphology, data requirements, input/outputs, and underlying algorithm.
2. Technical comparison: In-depth pros and cons for the six different types of emerging imitation learning approaches mentioned above, along with key related papers.
3. Deployment insights and current trends: Practical challenges for hardware implementation, including latency considerations and simulation-to-reality transfer strategies.

## 2 Taxonomy

Table 1 lists the notations used for categorizing each axis of the taxonomy, while Table 2 encodes each paper along the six orthogonal axes. Each axis is explained and justified in the following section, along with the categorization within each of them.

**TABLE 1:** Notation key for taxonomy.

Category	Notation and description
Data	A = Animal MoCap
	H = Human MoCap
	M = Expert MPC Logs
	T = Tele-operation or Virtual Reality (VR)
	V = In-the-wild Video Logs
	R = Robot Self-Logs
Technique	BC = Behavior Cloning
	AMP = Adversarial Motion Prior (GAIL)
	DIF = Diffusion-based Cloning
	DAP = Decaying/Latent Action Prior
	MPC = Hamiltonian/MPC-net Distillation
	CHRL = Curricular Hindsight RL
	MM = Mimic
Output	TQ = Joint Torques
	JP = Joint Positions
	TS = Task-space Wrench/GRF
Robot	Q = Quadruped
	B = Biped/Humanoid
	Hy = Hybrid (e.g., biped mode on quad chassis)
Deployment	S = Simulation Only
	RH-ind = Real Hardware, Indoor/Lab
	RH-out = Real Hardware, Outdoor/Field
Setting	OFF = Offline IL Only
	IL → RL = Imitation Learning Pre-training → RL
	IL + ADPT = Imitation Learning + Online Adaptation

**TABLE 2:** Taxonomy of imitation-learning papers for legged robots**.**

Paper and short title/Method	Demo	Tech	Out	Robot	Deploy	Setting
Ross et al. (2011)—DAgger: dataset-aggregation imitation	M	BC	JP	–	S	IL → RL
Peng et al. (2018)—DeepMimic: example-guided RL from human mocap	H	MM	JP	B	S	IL → RL
Peng et al. (2020)—AMP agile Laikago from animal MoCap	A	AMP	JP	Q	RH-ind	IL + ADPT
Lee et al. (2020)—ANYmal rough-terrain BC	M	BC	JP	Q	RH-out	OFF
Carius et al. (2020)—MPC-net	M	MPC	TQ	Q	RH-ind	OFF
Reske et al. (2021)—MPC-net multi-gait cloning	M	MPC	TQ	Q	RH-ind	OFF
Kumar et al. (2021)—RMA: BC + rapid adaptation	R	DAP	JP	Q	RH-out	IL + ADPT (hybrid IL + RL)
Escontrela et al. (2022)—AMP: adversarial motion priors quadruped	A	AMP	JP	Q	RH-ind	OFF
Yao et al. (2022)—Consistency video IL + adaptation	V	AMP	JP	Q	RH-ind	IL → RL
Miki et al. (2022)—Robust perceptive loco (outdoor)	M	BC	JP	Q	RH-out	IL + ADPT
Ajay et al. (2022)—Decision diffuser: trajectory-diffusion offline RL	R	DIF	JP	–	S	OFF
Khadiv et al. (2023)—Sensor-space BC of MPC expert	M	BC	JP/TQ	Q	S	OFF
Seo et al. (2023)—Tele-op humanoid loco-manip	T	BC	TQ	B	S	OFF
Ding et al. (2023)—SAF-BC task-space biped gaits	M	BC	TS	B	RH-ind	OFF
Yang et al. (2023)—semantics-aware locomotion from human demos	T	BC	JP	Q	RH-out	OFF
Sood et al. (2023)—DecAP: decaying action priors	R	DAP	TQ	Q	RH-ind	OFF
Vollenweider et al. (2023)—Multi-AMP skill library	A	AMP	TQ	Q	RH-ind	OFF
Huang et al. (2024)—DiffuseLoco: diffusion-BC quad	R	DIF	TQ	Q/B	RH-ind	OFF
Serifi et al. (2024)—RobotMDM: text-conditioned diffusion	H	DIF	JP	B	RH-ind	OFF
He et al. (2024)—Visual loco-manip IL	M	DIF	JP	Q	RH-ind	OFF
Song et al. (2024)—Differentiable-sim BC (quad)	M	BC	JP	Q	S	OFF
Peng et al. (2024)—AMP: biped walk using quad framework	M	AMP	JP	Hy	S	OFF
Mothish et al. (2024)—BiRoDiff: diffusion policies for unseen terrain	R	DIF	JP	B	S	OFF
Qiu et al. (2024)—WildLMa long-horizon loco-manip	T	BC	TQ	Q	RH-out	IL + ADPT
Hausdörfer et al. (2024)—1-cycle latent action priors	H	DAP	JP	Q	S	OFF
Li et al. (2024a)—OKAMI: single video humanoid manipulation	V	BC	JP	B	RH-ind	OFF
Li et al. (2024b)—CHRL: curricular hindsight RL	R	CHRL	JP	Q	RH-out	OFF
Zhang et al. (2024)—Humanoid walking w/human reference	H	AMP	JP	B	RH-ind	OFF
Narayanan et al. (2025)—GROQLoco: dataset-driven quad BC	R	BC	JP	Q	RH-out	OFF
Sajja et al. (2025)—Multi-task IL from NMPC logs	M	MPC	JP	Q	RH-ind	OFF
Shi et al. (2025)—ALMI-AMP	H	AMP	JP	B	RH-ind	OFF
Sood et al. (2025)—APEX: decaying action priors	A	DAP	JP	Q	RH-ind	OFF
Zhang et al. (2025)—Motion Priors Re-imagined	A	BC	JP	Q	RH-out	IL → RL
Ze et al. (2025)—TWIST: whole-body teleop imitation (humanoid)	H	BC	JP	B	RH-ind	IL → RL
Ma et al. (2025)—StyleLoco: GAN-distilled natural humanoid	H	AMP	JP	B	RH-out	IL → RL
Niu et al. (2025)—Human2LocoMan: cross-embodiment quadruped	H	BC	JP	Q	RH-ind	OFF

### 2.1 Data source

Expert data quality is the single strongest predictor of sim-to-real success in imitation learning for legged locomotion. Demonstration data sources have expanded beyond traditional motion capture to encompass teleoperation logs, massive robot trajectory datasets, and raw video footage from online platforms. The effectiveness of imitation learning depends critically on the quality and source of demonstration data, with current approaches utilizing six primary data sources that present unique trade-offs between data quality, availability, and morphological similarity to the target robot.

Animal MoCap (A) appears in five papers (14%) and exposes naturalistic gait phasing—the foundation of agile skills in Laikago (Peng et al., 2020) and the dog-sized quadruped Go-1 (Singla et al., 2019). This approach offers high-fidelity kinematic data but is typically constrained to laboratory environments and lacks the diversity needed for robust outdoor locomotion.

Human MoCap (H) represents eight papers (23%) and seeds bipedal balance in humanoids (Zhang et al., 2024; Taylor et al., 2021). Human demonstration data offer the advantage of abundant, diverse locomotion patterns readily available from internet-scale datasets. However, this approach faces significant embodiment gaps due to differences in morphology, joint configurations, and mass distributions between humans and legged robots.

MPC logs (M) dominate with 11 papers (31%) and are scalable, noise-free, albeit domain-limited. Policy execution from existing controllers—from both simulation and the robot—provides morphologically consistent data, but this approach may be limited in behavioral diversity.

Tele-operation (T) appears in three papers (9%) and contributes highly diverse but inconsistent contact patterns (He et al., 2024; Qiu et al., 2024). Robot teleoperation provides morphologically consistent data but is limited by the complexity of capturing full-body locomotion patterns and the substantial effort required for data collection.

Video (V) recordings are utilized in two papers (6%) and provide abundant, diverse locomotion data from internet-scale sources at low cost, capturing natural behaviors without expensive motion capture equipment or specialized environments. However, significant embodiment gaps between video subjects and robots create complex morphological mismatches, while extracting actionable robot control data from visual observations requires sophisticated computer vision and physics-informed processing techniques. Robot self-logs (R) appear in six papers (17%) and scale to thousands of trajectories but carry severe covariate shift, along with video data. Both video and robot self-logs offer unprecedented scalability but require addressing technical challenges related to embodiment gaps and context translation.

### 2.2 Imitation learning technique

As mentioned in Section 1, IL for legged robot locomotion now spans a spectrum of method families—including BC, AMPs, DIFs, DAPs, Hamiltonian and MPC variants (MPCs), and CHRL—each differing in their use of supervision, generative or adversarial regularization, and physical structure preservation. BC overwhelmingly dominates with 15 papers (43%), minimizing supervised loss in a hardware-friendly manner but remaining sensitive to covariate drift, which arises from the difference in the distribution of states visited by a learned policy during deployment, as compared to training. AMP appears in eight papers (23%), leveraging discriminators to enforce realism and often combining multiple priors for robust, style-rich motion. DIF represents five papers (14%), using stable denoising diffusion models for multimodal action synthesis. MPC methods account for three papers (9%), imposing structure or safety constraints through Hamiltonian imitation or MPC distillation to ensure physical feasibility. DAP methods also appear in three papers (9%), interpreting teacher actions as priors that decay across time to support practical torque-space deployment, and CHRL represents the smallest category with one paper (3%), utilizing IL as a curriculum seed for more adaptive, hindsight-driven reinforcement learning. This distribution reveals BC’s overwhelming practical dominance (representing almost half of all surveyed work) while highlighting the field’s growing but still nascent exploration of more sophisticated approaches that address fundamental limitations through adversarial training, generative modeling, structured priors, and online adaptation mechanisms. These six categories define the main axes of current research and are explored more in later sections to clarify their trade-offs and deployment considerations.

### 2.3 Output interface

The control output representation fundamentally shapes deployment feasibility and performance characteristics in legged locomotion systems. Our survey reveals that approximately 75% of reviewed works generate joint position (JP) targets as their primary control output, reflecting the field’s preference for kinematic-level commands. The position-level control approach has gained considerable traction, particularly in humanoid robotics research, by deliberately avoiding the intricacies of motor dynamics and hardware-specific control loops. This abstraction significantly simplifies the sim-to-real transfer process by delegating actuator-level concerns to the robot’s native control stack, although it may compromise some degree of fine-grained force control and dynamic responsiveness.

Raw joint torques (TQs) represent a more direct but challenging approach, offering maximum expressiveness and enabling precise force modulation essential for contact-rich locomotion. Although torque-level control allows seamless integration into whole-body control architectures, it demands robust sim-to-real transfer to handle actuator dynamics, sensor noise, and hardware limitations effectively.

Task-space wrenches (TS)—encompassing end-effector forces and ground reaction forces—have emerged prominently in safety-critical applications and curricular hindsight reinforcement learning pipelines. This renewed interest in force-controlled legged locomotion reflects growing recognition that explicit force reasoning can enhance robustness, safety, and adaptability. By operating in task-space coordinates, these approaches can more naturally incorporate physical constraints, contact force limits, and stability margins, making them particularly valuable for applications requiring predictable interaction forces with the environment or human operators.

### 2.4 Robot morphology

The distribution of robot platforms in legged-locomotion imitation learning reflects both technological maturity and emerging research frontiers. Analyzing the surveyed works reveals clear patterns in morphology preference and deployment environments that highlight the field’s current capabilities and limitations.

Quadrupedal (Q) platforms overwhelmingly dominate the literature, appearing in 24 of the surveyed papers, reflecting their inherent stability advantages and the relative maturity of four-legged control frameworks. This strong preference stems from the natural redundancy of quadrupeds in ground contact, which provides greater tolerance for control errors and simplifies the sim-to-real transfer process. Popular platforms include the ANYmal series, Laikago, and Unitree Go-1 robots, with applications ranging from rough terrain navigation to agile locomotion skills derived from animal motion capture data.

Bipedal and humanoid platforms (B), while representing only nine papers, are experiencing rapid growth, with particularly strong momentum, in post-2023 research. This surge is exemplified by recent works such as whole-body humanoid control using human motion references (Zhang et al., 2024), teleoperative humanoid locomotion and manipulation (Seo et al., 2023), and cross-embodiment imitation learning approaches (Niu et al., 2025). Despite being outnumbered by quadrupeds, the increasing interest in bipedal systems reflects growing confidence in handling their inherent dynamic complexity and the potential for more human-like robot behaviors. Hybrid configurations (Hy), although less common, represent an interesting middle ground where robots can switch between quadrupedal and bipedal modes depending on task requirements.

### 2.5 Deployment

The deployment distribution reveals significant challenges in real-world application of imitation learning methods. Simulation-only work (S) accounts for 8 out of 35 papers (23%), providing a safe testing ground for new algorithms while highlighting ongoing sim-to-real transfer difficulties. The majority of work focuses on indoor laboratory deployments (RH-ind), where controlled conditions enable reliable reproduction of learned behaviors. Critically, outdoor hardware deployment (RH-out) appears in only 8 out of 35 surveyed works, underscoring the substantial gap between laboratory demonstrations and field-ready systems.

### 2.6 Learning setting

The surveyed literature reveals three distinct paradigms for handling the learning process in legged-locomotion imitation learning. Pure offline imitation learning (OFF) is predominant, appearing in 25 out of 35 papers (71%), where robot policies are trained entirely on pre-collected demonstration data without any subsequent environmental interaction. This approach learns from fixed expert datasets and deploying without further learning. The prevalence of offline-only methods reflects the field’s emphasis on predictable, controlled learning environments.

However, a notable shift toward adaptive learning paradigms is emerging through two hybrid approaches. IL → RL (six papers) follows a sequential two-phase pipeline where imitation learning provides initialization followed by reinforcement learning that enables skill discovery and environmental adaptation through continued interaction. IL + ADPT (three papers) maintains a stable base policy trained on demonstrations while incorporating concurrent online adaptation mechanisms that make real-time adjustments during deployment based on sensory feedback. The growing adoption of these hybrid frameworks—representing nearly 26% of surveyed works—indicates field-wide recognition that pure offline methods, while safe and stable, may be insufficient for the robustness demands of real-world legged locomotion, driving a gradual evolution toward longer-duration learning paradigms that can continuously adapt to new environments and conditions.

## 3 Imitation learning methods

This section gives the details of each of the six categories of the algorithms mentioned earlier and listed paper-wise in Table 2. The main papers for each category are also discussed.

### 3.1 Behavior cloning

Despite its simplicity, BC remains a foundational approach in imitation learning for legged robots. It offers a straightforward supervised learning framework, where the objective is to train a policy $\pi_{\theta }\left(a_{t}|s_{t}\right)$ to map observed states $s_{t}$ directly to expert actions $a_{t}$ by minimizing the discrepancy between the robot’s actions and those of a demonstrator. Given a dataset of expert trajectories $D={\left\{s_{t}^{i},a_{t}^{i}\right\}}_{i=1}^{N}$ , the standard BC objective is to minimize the empirical risk

$$L_{\text{BC}}\left(\theta \right)=\frac{1}{N}\overset{N}{\underset{i=1}{\sum }}\ell \left(\pi_{\theta }\left(s_{t}^{i}\right),a_{t}^{i}\right)$$ (1)

where ℓ(.,.) is typically the mean squared error (MSE) for continuous actions or cross-entropy for discrete actions (Ross et al., 2011), $\pi_{\theta }\left(s_{t}^{i}\right)$ is the predicted action, $a_{t}^{i}$ is the expert action, and N is the total number of expert trajectories.

Three design choices govern BC performance: demonstration fidelity, state augmentation, and feedback tracking. Sensor-space cloning (Khadiv et al., 2023) achieves 400 Hz torque control by ingesting only proprioception; no external vision is required for flat terrain. For cases where vision is critical, He et al. (2024) combined RGB-D with foot force sensors to execute door-pushing while trotting. Although BC provides a data-efficient and hardware-friendly pathway to policy learning, it is fundamentally limited by its sensitivity to covariate shifts. When a BC system encounters states outside the training distribution, errors can compound quickly over time steps, leading to catastrophic failures, as demonstrated by Ross et al. (2011).

Recent research (Kumar et al., 2021; Qiu et al., 2024) addresses these shortcomings by combining BC with online adaptation layers or by augmenting demonstration datasets with greater diversity and domain randomization. As a result, BC continues to be a practical baseline for researchers and can be a critical component if one chooses to create a more sophisticated hybrid system around it.

In their work, Narayanan et al. (2025) (GROQLoco) extended their approach to multi-terrain logs, using domain randomization to narrow the sim-to-real gap and achieving an 85% success rate on rubble. They developed a single, generalist locomotion policy capable of handling various quadrupedal robots across diverse terrains. They achieved this by training on expert demonstrations that include both stair and flat terrain traversal, leveraging data gathered from several quadrupeds to encompass a wide range of gaits and morphological diversity. The central argument of their study is that enhancing diversity in both robot body types and locomotion behaviors is essential for achieving robust generalization. To validate this, they collected data using multiple quadruped robots operating on stairs and flat surfaces. Their generalist policy was then deployed on platforms such as the Unitree Go-1 and Stoch-5, without requiring any additional fine-tuning steps. The model architecture features causal attention mechanisms, alongside GRU-based temporal modeling to effectively capture the dynamics of locomotion across these varied settings.

Seo et al. (2023) introduced a framework Tele-Operation and Imitation Learning for Loco-Manipulation (TRILL), which deals with training humanoid loco-manipulation policies using human demonstrations by using a virtual reality (VR) tele-operation interface to collect human demo data. For humanoid robots, since the task action space is vast, the dataset is also enormous, leading to slow training rates. A second challenge is in terms of dealing with contact-rich environments and the need for stabilizing dynamics. They used a whole-body control approach to convert the task-space trajectories into joint-torque actions and implemented policies for humanoid bimanual operation tasks, such as picking and placing and removing a spray cap. The main challenge noted in their work is the control latency, which makes it more difficult to transfer the policies to different hardware.

Qiu et al. (2024) presented a comprehensive structure for combining whole-body control, imitation learning, and the use of Large Language Models (LLMs) for the planning of manipulation of a quadruped. They used VR tele-operation to collect data. The method also develops a generalizable skill library of visuomotor skills using imitation learning and analytical methods (such as way-point navigation using PD-based control and LiDAR-based SLAM for pose estimation). Finally, there is a task planning system interfaced with LLMs that can decompose a high-level command into small individual tasks. They deployed the controller on a Unitree B1 quadruped with a Z1 arm for applications related to table top grasping, button pressing, and grasping from ground. They also showed some long-horizon tasks such as trash collection and shelf rearrangement. The main limitations were that the success rate of the long-range tasks was moderate, showing the need for error recovery mechanisms.

Yang et al. (2023) presented a framework that enables quadruped robots to adapt their locomotion behaviors based on terrain semantics (e.g., grass, mud, and asphalt) rather than only geometric properties. The key innovation is learning directly in the real world using only 40 min of human demonstration data while maintaining safety and efficiency. They use tele-operated data across diverse terrains collected using a human operator using joystick commands. The high-level skill policy selects the locomotion gait and speed from camera images, while a low-level MPC controller is used for motor commands. They show the policy being deployed on the Unitree A1 quadruped on a 450 outdoor trail. The learned policy is able to run on near-maximum safe speeds on asphalt, grass, pebble, and rock surfaces. The policy’s main limitation is it’s inability to perform agile movements such as jumping. It can also reflect the human operator’s cautiosness by behaving in an overly conservative manner.

Teleoperated Whole-Body Imitation System (TWIST) (Ze et al., 2025) presents a method for humanoid tele-operation that enables real-time whole-body motion imitation. Unlike the traditional approach that decouples upper and lower body control or focuses on isolated tasks, TWIST achieves coordinated whole-body skills through a unified neural network controller. TWIST uses a three-stage pipeline, with the first stage focused on humanoid motion dataset curation using MoCap clips from AMASS and OMOMO datasets. Next, a teacher–student policy is trained using proximal policy optimization (PPO), where the teacher has privileged access to 2-s future motion frames. Finally for the last stage, Optitrack MoCap is used to perform real-time re-targeting for humanoid motion generation, with a loop rate of 50 HZ for the joint targets. They deployed the policy on the Unitree G1 and the Booster T1 (for sim-to-sim validation) for whole-body manipulation skills, such as lifting boxes and carrying objects. They also showcased legged manipulation, such as kicking soccer balls and opening doors with feet. The main limitations, like other research in this area, are tele-operation delay hindering real-time critical tasks, with no tactile feedback.

Object-aware Kinematic retArgeting for huManoid Imitation (OKAMI) (Li J. et al., 2024) presents a breakthrough for teaching humanoid robot manipulation skills from single-RGB-D video demonstrations. The key innovation is object-aware re-targeting, which enables robots to mimic human motions while adapting to different object locations during deployment. They presented a two-stage training pipeline, where the first stage deals with the reference generation, followed by SLAM in the second stage. They integrated the GPT4V model to identify task-relevant objects and use the modified SLAHMR model with the SMPL-H model for full body and hand poses. Stage-2 is based on object-aware re-targeting based on ground SLAM. Finally, they applied inverse kinematics to convert re-targeted trajectories to joint commands. They deployed the controller on a Fourier GR1 humanoid with 6-DoF dexterous hands for tasks such as placing snacks on plates, closing the laptop, closing the drawer, and bagging. However, the pipeline only supports manipulation and no locomotion, and the performance is inconsistent with motion speed and quality.

Niu et al. (2025) introduced a novel cross-embodiment imitation learning framework that enables quadrupedal robots to learn manipulation skills from human demonstrations. The system’s core technical innovation lies in its Modularized Cross-embodiment Transformer (MXT) architecture, which uses separate tokenizers and detokenizers for different data modalities while sharing a common transformer trunk across embodiments. The cross-embodiment learning capability is particularly noteworthy, achieving a 38.6% success rate improvement through human pretraining and showing strong positive transfer, despite the large morphological gap between humans and quadrupeds. There certainly are scalability questions regarding how effective the approach is when handling larger-scale datasets or more diverse robot embodiments. The system’s generalization to other quadrupedal platforms remains invalidated.

### 3.2 Adversarial motion priors

AMPs represent a class of imitation learning methods based on generative adversarial imitation learning (GAIL) (Ho and Ermon, 2016) by leveraging a discriminator to regularize policy learning, ensuring that generated motion remains realistic and closely aligned with expert demonstrations. The policy is trained within an adversarial framework: a discriminator network is tasked with distinguishing between state–action pairs from the expert dataset and those produced by the policy, while the policy aims to produce behaviors that the discriminator cannot differentiate from those of the expert. AMP methods are particularly effective in capturing the style and naturalness of motion, which is crucial for legged locomotion. For example, Peng et al. (2018) showed in their DeepMimic framework that adversarial objectives are able to produce highly dynamic and agile motions in simulated humanoids.

AMP’s discriminator inherits the reward-design burden: if it is too weak, the policy diverges; if too strong, learning collapses. Lee et al. (2020) mitigated this by expanding the teacher dataset to 7 k MPC trajectories, achieving 1.5 m/s over gravel, while Vollenweider’s multi-prior variant (Vollenweider et al., 2023) blends gait styles (trot, bound, and jump) into a single policy with 92% automatic mode-selection accuracy. The hybrid biped-on-quadruped demo (Peng et al., 2024) underscores AMP’s robustness to morphology mismatch.

The discriminator $D_{\phi }\left(s,a\right)$ tries to distinguish between state–action pairs from reference motion and those generated by the policy $\pi_{\theta }\left(a|s\right)$ . The task of the policy is to “fool” the discriminator while maximizing any task-specific reward. The AMP objective augments the standard RL objective with an adversarial imitation term:

$$\max_{\theta }\;E_{\pi_{\theta }}\left[r\left(s,a\right)+\lambda \log D_{\phi }\left(s,a\right)\right]$$ (2)

where r(s,a) is the environment or task reward, $D_{\phi }\left(s,a\right)$ is the discriminator’s output, and λ is a weighting factor that balances task performance and motion realism. The discriminator itself is trained on the ability to maximize its ability to distinguish expert from policy-generated data

$$\max_{\phi }\;E_{\left(s,a\right)∼\text{Expert}}\left[\log D_{\phi }\left(s,a\right)\right]+E_{\left(s,a\right)∼\pi_{\theta }}\left[\log\left(1-D_{\phi }\left(s,a\right)\right)\right]$$ (3)

This is analogous to the discriminator loss in GANs, where the goal is to correctly classify real (expert) versus fake (policy) samples (Goodfellow et al., 2014). The policy is updated using RL (e.g., PPO or SAC), where the reward at each step is augmented by the discriminator’s output, encouraging the policy to generate expert-like motions. In practice, state s often includes proprioceptive features (joint angles, velocities, and base orientation) and sometimes exteroceptive features (terrain and vision), while action a is typically joint positions or torques. Overall, AMP has shown to produce more natural, energy-efficient, and robust gaits than pure RL, especially when rewards are sparse or under-specified (Merel et al., 2017).

Peng et al. (2020) combined the motion imitation from animals, along with latent space adaptation to learn a diverse set of dynamic locomotion skills, which are ultimately transferred to quadrupeds. Their pipeline is divided into motion re-targeting, motion imitation, and domain adaptation. Although motion re-targeting is often performed using inverse kinematics solvers, motion imitation is performed by training a policy in simulation using domain randomization. Finally, the policy is transferred to real robots using the sample-efficient domain adaptation process. The policy is queried at 30 Hz for a new action at each time-step. The action space specifies joint positions for PD controllers at each joint, after being low-pass-filtered. The motion dataset consists of MOCAP clips from dogs and some from artist animations.

Escontrela et al. (2022) highlighted that standard RL approaches can yield aggressive, overly energetic behaviors due to under-specified rewards. To address this, they used motion capture data to create a “style-reward,” encouraging agents to mimic the style of reference motions. This method leads to lower cost-of-transport (CoT) and more natural gait transitions. Similarly, Zhang et al. (2024) used an AMP-based imitation learning framework with a motor-joint-driven humanoid, Adam, trained via PPO in Issac Gym. They successfully demonstrated human-like, straight-knee “heel-to-toe” gaits.

Peng et al. (2024) discussed an AMP-based approach to adapt a learning framework designed for quadrupedal motion to operate on bipeds. This allows them to use the front two legs to perform useful work, while using the hind legs for locomotion. They followed the approach of a student–teacher policy to enable imitation learning using reference motion. For reference generation, they used the TOWR (Winkler et al., 2018) library to perform trajectory optimization (TO) for the A1 biped robot. This results in dynamically and kinematically feasible reference trajectories that can be used for learning. The teacher policy uses a PPO algorithm using the Issac gym simulator. They tested the policy in simulation on different terrains, such as uniform, wave, stepping stones, sloped, stairs, and obstacles. The policy performed well on lower speeds and gradually worsened on higher speeds and sloped terrain, with obstacles.

The VIAN framework (Yao et al., 2022) enables quadrupeds to mimic animal behaviors from brief videos (3–8 seconds) using deep RL guided by consistency-based rewards. It uses DeepLabCut (Nath et al., 2019) for pose estimation, mapping key anatomical points from animals to robots. VIAN handles both periodic (e.g., walking) and aperiodic (e.g., backflip) motions, adapting motions through seasonal decomposition for periodic gaits and keyframe selection for aperiodic gaits. Trained in PyBullet and deployed on the A1 quadruped, VIAN achieved an 80% success rate for dog imitation versus 55% for standard RL, highlighting the strength of video imitation.

StyleLoco (Ma et al., 2025) introduced a Generative Adversarial Distillation (GAD) framework that overcomes the trade-off in humanoid locomotion between agility and naturalness. It uses two discriminators: a teacher discriminator ensures that the student policy maintains RL-derived agility and precision, while a dataset discriminator enforces natural movements by referencing human motion-capture data (LaFAN1). Tested on the Unitree H1 in simulation and real-world settings, StyleLoco achieves agile, robust, and natural human-like walking. The main limitation is the need for manual tuning of discriminator weights.

Multi-AMP (Vollenweider et al., 2023) extends the AMP framework, enabling robots to learn and seamlessly switch between multiple motion styles within a single policy. A key highlight is its demonstration on a wheeled-legged quadruped robot, which can perform advanced skills such as quadruped-to-humanoid transformation: standing upright on its hind legs, navigating on two wheels, and returning to a seated position. The architecture uses a dedicated discriminator for each motion style, with each discriminator solving a least-squares task to distinguish real motion data from policy-generated actions. Training occurs in Issac Gym on a 16-DoF wheeled-legged quadruped, showing diverse behaviors, including standard four-legged movement, ducking under obstacles, and morphing between quadruped and humanoid gaits. The main limitations include the need to generate motion data for most skills and the challenge of tuning multiple discriminators for stable learning.

### 3.3 Diffusion cloning

Diffusion cloning refers to a new type of imitation learning paradigm that uses denoising diffusion models (originally developed for image synthesis) to learn a set of robust robot control policies from demonstration data. Instead of mapping states directly to actions, a diffusion policy gradually refines a random initial action toward a realistic, expert-like action by iteratively denoising over multiple steps, guided by context (such as images or language) (He et al., 2019).

These models are trained on offline demonstration data and have shown strong generalization and robustness to out-of-distribution scenarios compared to standard policies. Mani et al. (2024) and Serifi et al. (2024) showed that modern variants can condition on high-dimensional vision features and even plain text instructions for language-conditioned behavior.

Starting with an expert action $a_{0}$ from demonstration, the action is progressively perturbed over T steps with Gaussian noise:

$$a_{t}=\sqrt{{\bar{\alpha }}_{t}a_{0}}+\sqrt{1-{\bar{\alpha }}_{t}\epsilon }$$ (4)

where ϵ∈(0,1) and $\alpha_{t}$ control the noise schedule (typically decreasing over time). The policy learns to denoise: recover $a_{t-1}$ from a , conditioned on state s (and often context, such as images or text). The denoising neural network predicts the noise ϵ added at step t .

$${\hat{\epsilon }}_{\theta }\left(a_{t},\;s,\;t,\;c\right)$$ (5)

The training loss is typically a mean squared error (MSE) between real noise ϵ and predicted noise ${\hat{\epsilon }}_{\theta }$

$$L=E_{a_{0},\;\epsilon ,\;t}\left[{\left\|{\hat{\epsilon }}_{\theta }\left(a_{t},s,t,c\right)-\epsilon \right\|}^{2}\right]$$ (6)

To generate a new action, we start from pure noise $a_{T}$ and iteratively apply the learned denoising model T times (for t=T,…,1 ), updating $a_{t}$ toward $a_{0}$ :

$$a_{t-1}=f\left(a_{t},s,t,c,{\hat{\epsilon }}_{\theta }\right)$$ (7)

where f denotes the standard diffusion model update (can be model-specific).

It gets expensive to scale RL training due to expensive rollouts. DiffuseLoco (Huang et al., 2024) attempts to solve this problem by denoising over a 16-step horizon at 100 Hz on Jetson Orin, outputting torques. They solved two problems—offline learning from various data sources and the ability to learn a set of diverse skills by training diffusion-based policies that capture diverse behaviors, enabling learning in both quadrupedal and bipedal settings. They were also able to generate plans higher than 30 Hz. Their state-space is modeled as the effector’s proprioceptive feedback-measured joint positions q , joint velocities $\dot{q}$ , base orientation Θ , and base angular velocity Ω . Their action space is the desired joint position, while goal space is the desired base height, sagittal velocity, and desired turning velocity. The method was also deployed on the bipedal Cassie robot but exhibited poor sim-to-real tranfer compared to the Go1 quadruped example. The DiffuseLoco policy demonstrates good robustness against various ground conditions and small variations in terrain. However, robustness against a specific skill is poor.

Mothish et al. (2024) trained a single walking controller that yields locomotion on multiple terrains. Their BiRoDiff biped controller reaches 0.85 m/s on 15 slopes purely in sim, and hardware transfer depends on real-time inference optimization. The training is based on the diffusion model, generalizes on multiple terrains, and uses offline data. RobotMDM (Serifi et al., 2024) introduces text tokens (e.g., “*low crouch*”) at every denoising iteration, gesturing toward language-grounded locomotion. They used a two-stage process, where they first trained a Critic model from a dataset, creating a differentiable surrogate for expected future rewards conditioned on motion inputs. For the second stage, the Critic is used to fine-tune a diffusion model to align with the character’s limits and physical feasibility. For training purposes, they used the HumanML3D dataset (Guo et al., 2022), consisting of the human motion-capture data, re-targeted for the bipedal robot character. However, the main limitation remains the lack of hard constraints on motion feasibility, hence limiting its use in performance-critical tasks. They recommended the use of physics-aware motion generators to create new datasets for training.

He et al. (2024) presented a hierarchical RL-based controller and a behavior cloning planner for a quadruped to perform loco-manipulation. The high-level planning policy is based on the diffusion-based BC approach. The main benefit of the method is that they can carry out locomotion while performing any manipulation task. The fundamental approach deals with collecting data using a low-level control policy for the end-effector to follow Bezier control points while maintaining locomotion using the three remaining legs. The manipulation end-effector trajectory is parametrized, and the parameters are outputs of the high-level planner. The large-scale datasets are collected using parallel simulation in IssacGym (Makoviychuk et al., 2021). They can perform tasks including pressing a button, pulling handles, pushing doors, and opening a dishwasher and achieve better success rates than the hierarchical reinforcement learning method. The main limitations are inference speed limitations and poor sim-to-real transfer.

Decision diffusers (Ajay et al., 2022) introduced a diffusion probabilistic model to generate high-quality trajectories by conditioning on returns, constraints, or skills, eliminating the need for value function estimation. They presented a single approach that handles return maximization, constraint satisfaction, and skill composition through different conditioning strategies. The approach is mostly applied in simulation for locomotion tasks on HalfCheetah, Hopper, and Walker2D. They also showed some long-horizon D4RL kitchen tasks, along with Unitree quadruped simulation. The main limitations reported are stochastic dynamics—where performance degrades in highly stochastic environments—and limited data regimes, which make the model prone to overfitting with small datasets.

### 3.4 Decaying/latent action priors

DecAP (Sood et al., 2023) addresses the fundamental challenge of learning torque-based locomotion policies for legged robots—more robust and compliant than position-based policies but suffer from sample inefficiency and poor convergence to natural gaits. They proposed a two-stage framework that leverages the sample efficiency of position-based learning to accelerate torque-based learning. The first stage trains an end-to-end joint position-based policy using PPO with standard locomotion rewards, along with collecting imitation data including joint angles, base height, and foot height. The second stage trains a torque-based policy using the data from the first stage. They introduced the Decaying Action Priors (DecAP), which are torque biases calculated on the joint angles via a PD controller. They showed that without any domain randomization, the torque policy maintains smooth outputs during perturbations, while the joint-position policy fails. The main limitations are that the framework depends on offline imitation data from position-based policy simulations. However, the framework requires manual tuning of the PID gains for the position-based policy. Eventually, the system transitions to a fully torque-based policy. DecAP reports 30% shorter training time than BC while halving torque overshoot in disturbance tests.

Hausdörfer et al. (2024) introduced latent action priors, a novel approach that learns compressed action representations from minimal expert demonstrations to guide deep reinforcement learning. The latent action prior method learns a low-dimensional latent representation of expert actions using an autoencoder. This latent prior guides the RL and improves the performance and generalization. $z_{t}=Encoder\left(a_{t}\right)$ and ${\hat{a}}_{t}=Decoder\left(z_{t}\right)$, where $z_{t}$ is the latent code and the reconstruction loss is

$$L_{\text{AE}}=\underset{t}{\sum }{\left\|a_{t}-{\hat{a}}_{t}\right\|}^{2}$$ (8)

They demonstrated that effective action priors can be extracted from only a single open-loop gait cycle, dramatically reducing data requirements while improving learning performance and enabling above-expert-level achievements. They used a nonlinear auto-encoder with one hidden layer, and the latent space dimension is set to half of the full action space. The policy is implemented in Loco–Mujoco using the Unitree A1 and H1 humanoids, along with the Mujoco environment for HalfCheetah, Ant, and Humanoid. They showed different gait transitions from walking to running to galloping across speed ranges.

Zhang et al. (2025) introduced a hierarchical RL framework that enables quadruped robots to generalize motion imitation skills from flat-terrain animal data to complex terrains by learning low-level motion priors and adapting with high-level residuals. Their four-step pipeline—motion processing, motion prior pre-training, hierarchical adaptation, and sim-to-real distillation—culminates in a real-world deployable policy on ANYmal-D using a GRU belief encoder for sim-to-real transfer. However, the method can suffer from mode collapse (defaulting to a single gait), does not support non-locomotion skills (such as jumping or crawling), and excludes highly discontinuous terrains (e.g., gaps or stepping stones).

Rapid Motor Adaptation (RMA) (Kumar et al., 2021) introduces a hybrid structure of supervised learning for the adaptive module with reinforcement learning for the base policy. It is a transformative framework for enabling real-time adaptation in quadruped robots, allowing them to traverse a wide range of challenging terrains without requiring simulation calibration or additional fine-tuning in the real world. They introduced a two-part system: a base policy, initially trained using privileged (environment-specific) information, and an adaptation module that dynamically estimates environmental factors by analyzing recent state-action histories. Training proceeds in two stages: first, the base policy is optimized via PPO using privileged data about the environment; then, a separate adaptation model is trained to infer critical environment parameters based on the last 50 steps of the robot’s own states and actions. RMA demonstrates deployment on the Unitree A1 quadruped for both indoor and real-world outdoor experiments, successfully navigating terrains such as sand, mud, grass, and irregular construction sites filled with pebbles and cement debris. A key limitation of this approach is its reliance solely on proprioceptive data, without utilizing external sensors or exteroceptive cues. However, it is important to note that RMA is not a purely imitation-based approach. Although the adaptive module is trained in a supervised imitation method to infer environment parameters from historical observations, the base locomotion policy itself is optimized with reinforcement learning using privileged information. This hybrid mechanism distinguishes RMA from pure imitation learning methods such as behavior cloning.

### 3.5 Hamiltonian and safety variants

Sajja et al. (2025) used expert demonstrations from non-linear model-predictive control (NMPC) to train a single neural network policy and to generalize the single policy on diverse quadrupedal gaits. They used raw proprioceptive data including IMU and joint-encoder measurements. A single neural network maps raw proprioceptive data to joint-position targets, and the outputs of the network are task-specific, one for each different type of gait (trot, bound, etc.). However, the model did not generalize well to new gait such as gallop or pace, showing limitations of multi-task learning. Similarly, Khadiv et al. (2023) also used NMPC demonstrations as an expert to learn policies directly from the proprioceptive data. They were able to learn different gaits on the solo-12 quadruped. They also showed that the joint-position target policy outperforms the torque policy. The architecture consists of two networks—an estimator network that maps measurements to states and a policy network that maps measurements to actions. They used Pybullet simulation environment for collecting the datasets by perturbing the system at each re-planning stage of the NMPC. They were able to show that the PD policy was superior to the torque policy since it is more robust to the function approximate error.

MPC-Net (Carius et al., 2020) presents a novel imitation learning approach that distills MPC solutions into fast neural network policies. The key innovation is using a theoretically motivated loss function based on the control Hamiltonian rather than traditional behavioral cloning, enabling robots to learn complex control policies from minimal MPC demonstration data while maintaining constraint satisfaction. They used a Hamiltonian-based loss function and a linear–quadratic controller as the expert demonstrator. The samples used for training are extracted from neighborhoods of the optimal trajectories. They also used a Gaussian sampling to create tubes of state-space as training data. The main advantage is the constraint-aware learning, which maintains physical feasibility. They showed the policy on the ANYmal quadruped robot for trotting and static walk gaits. The main limitations are that the resulting policy cannot outperform MPC for the same cost function and cannot learn in the areas where MPC does not converge.

Reske et al. (2021) presented training a single policy that learns multiple gaits of a walking robot. They used the mixture of experts network, where each expert is responsible for controlling one mode of a hybrid system. The core idea is to use a single policy to replace the teacher to control multiple gaits by distilling nonlinear MPC into a neural network, cutting compute by 20 times and enabling 1 kHz joint-space control on ANYmal.

Peng et al. (2020) used differentiable simulation for learning quadruped locomotion. Their work demonstrates that by using a differentiable simulation, they can outperform an RL PPO algorithm in terms of sample efficiency, handling large-scale environments. The main approach is to first split the robot-dynamics model into a floating base and joint space. They avoided using the full of the whole-body model that has discontinuities due to contact models and used the single-rigid-body model instead. For training the policy, they used the simple surrogate model and the full non-differentiable simulator for the forward simulation. They deployed the policy on a Mini-Cheetah for different gaits (trot, pace, bound, and gallop) on challenging terrains.

### 3.6 Curriculum hindsight reinforcement learning

Although CHRL is framed as a reinforcement learning approach, it incorporates a teacher–student policy architecture that bears conceptual similarities to imitation learning. In contrast, CHRL trains a teacher policy with privileged information (e.g., ground-truth terrain, friction, and payload) and then distills its behavior into a student policy with only proprioceptive inputs. This distillation process resembles IL in that the student learns to mimic the teacher’s actions under limited observations; the teacher is an RL-trained agent and not a human expert. We, therefore, decide to include CHRL in this review because its teacher–student framework highlights a broader class of methods, where knowledge transfer from privileged to non-privileged policies plays a role similar to imitation. This situates CHRL at the intersection of imitation learning and reinforcement learning with privileged information.

Li et al. (2024b) introduced CHRL, a novel framework that enables quadruped robots to achieve highly agile and adaptive locomotion behaviors including fall recovery, high-speed running, and rapid turning in real-world environments. The strategy is an adaptive curriculum that adjusts task difficulty based on policy performance, and the main curriculum parameters are as follows: a) reward coefficients—joint torque penalties and energy costs; b) domain randomization on friction and payload mass; c) command ranges—linear velocities; and d) terrain difficulty—height field variations. For the learning architecture, they used a teacher–student policy architecture, where the teacher policy has access to the full privileged information (ground truth terrain, friction, and payload) and outputs a 12-dimension joint position targets. The student policy has access to only the proprioceptive sensors, along with added noise. They showed the policy on a custom quadruped in outdoor grass terrains with high forward/turning speeds. The main limitations are the usage of only proprioceptive sensors and careful tuning of curriculum thresholds that could be tedious.

### 3.7 Mimic

Although adversarial motion prior methods use discriminators to enforce motion realisim, DeepMimic (Peng et al., 2018) presents a deep reinforcement learning framework for physics-based character animation that combines motion imitation objectives with task-specific goals without the use of discriminators. The framework enables simulated characters to learn robust control policies that can reproduce a wide range of motion clips while adapting to environmental variations and accomplishing user-specified objectives. The state-features include character body configuration (link positions, rotations, and velocities) in the local coordinate frame, while the action space includes the target joint positions. The inputs to the model are reference motion capture clips (humans, animals, and key framed). The policy/neural network outputs joint positions, which are fed to PD controllers. PPO is used with reference state initialization and early termination to stabilize training. The rewards consist of a weighted combination of pose-reward, velocity reward, end-effector, and a center-of-mass reward. For training, the initial states are sampled from reference motion, rather than a fixed starting position. They showed more than 30 skills, including locomotion, martial arts for the Atlas robot, T-Rex, and dragon. DeepMimic offers high motion quality without significant reward engineering and handles dynamic acrobatic skills well. The main limitations are the PD controller tuning for each different character and the high sensitivity in novel states. The DeepMimic framework inspired many later works that directly clone MPC trajectories or animal gaits. It became a default baseline in legged locomotion before AMP, and diffusion-based approaches were adopted and are still widely used due to simplicity and robustness.

## 4 Deployment challenges

This section details some of the deployment challenges for the several imitation learning approaches discussed so far.

### 4.1 Data dependency

The data quality heavily impacts deployment success in most of the algorithms. As an example, VR teleoperation often introduces latency and tracking errors that propagate to learned policies, while human operators exhibit inconsistent performance, introducing suboptimal behaviors. There is performance drift due to facility modifications, evolving task requirements and seasonal variations. In some use cases, continuous demonstration collection for policy improvement requires on-going expert availability. In terms of data dependency, for behavior cloning methods, the covariate drift is a significant challenge during deployment. This drift happens when the system encounters a state outside the training distribution, leading to poor predicted action. The covariate drift also limits the policy not being able to discover more aggressive behaviors, as a result of human operators being more cautious during data collection. For AMP-based approaches, less time is spent on reward function tuning, compared to traditional RL approaches, where more time and effort are needed to generate quality motion priors for each targeted style, which might not always be feasible. Diffusion-based methods are prone to overfitting in case of limited data. This manifests as brittle policies that succeed in simulation but fail on hardware under slightly varied conditions (e.g., object slippage in manipulation and unstable balance in quadrupeds).

On the one hand, although simulation has become an effective data source for many robot learning tasks, modeling the complex contact dynamics accurately and rendering photorealistic terrains are not yet possible in simulation.

### 4.2 Sensor integration challenges

In most of the studies surveyed in this paper, a common theme was the use of multimodal sensor fusion. Sensor fusion brings complexity and often discovers new failure modes. Since IMU bias, camera intrinsics, and joint encoder offsets change over time, temporal alignment between the different sensor modalities becomes critical for stable performance. A common deployment failure is policy degradation due to small calibration drifts. An example of that would be quadrupeds mis-stepping when joint encoders slip by a few degrees or manipulation tasks failing due to depth-camera bias.

### 4.3 Predictability and interpretability

Papers surveyed here also revealed that black-box neural policies create deployment challenges for safety-critical applications. The inability to explain policy decisions complicates debugging and validation, while the lack of formal bounds on policy behavior under perturbations poses certification challenges for commercial deployment. For instance, a BC-trained manipulator may unexpectedly apply unsafe forces on fragile objects, but without interpretable mechanisms, it is difficult to anticipate or prevent such actions.

### 4.4 Sim-to-real strategies

Despite advances in domain randomization, zero-shot transfer often requires careful parameter tuning and may fail in scenarios significantly different from training distributions. A typical case is legged robots trained with randomized terrain slopes in simulation still failing to generalize to soft grass or snow, due to unmodeled compliance. For AMP methods, manual tuning of the discriminator is often required for stable learning. The latent representation shift across sim-to-real transfer poses fundamental limitations as observations that appear numerically similar may have completely different meanings in their respective contexts. This is especially problematic for diffusion-based methods, where latent mismatch can accumulate, producing actions that look smooth in simulation but destabilize real robots.

## 5 Future directions

Here, we list some key areas which are actively worked upon and play a key role in the future of imitation learning for legged locomotion.

Reducing the simulation-to-real gap: IL policies are usually trained in simulation for safety. Translating them to hardware often requires robust domain randomization, system identification, and actuator modeling. Contact-rich locomotion demands tactile and proprioceptive data, but existing sensors are limited. Future IL frameworks should incorporate diverse sensor-data modalities (joint positions, forces, vision, and tactile), better align human and robot perspectives, and integrate multimodal data to teach not only what movements to perform but also why.

Differentiable and high-fidelity simulators: Differentiable simulators allow gradient-based optimization of policies and promise better sample efficiency. However, legged locomotion involves stiff contact dynamics that can lead to poor local minima. Hence, continued work in smoothing techniques and improved contact models are needed. Research on differentiable simulation for IL could enable direct backpropagation through contact events and more efficient policy training.

Building benchmarks There is a lack of standard benchmarks for loco-manipulation tasks. However, learning benchmarks such as HumanoidBench (Sferrazza et al., 2024) and MimickingBench (Liu et al., 2024) provide initial test suites but need to be expanded. Future work should establish datasets and metrics for evaluating IL policies across gaits, terrains, and manipulation tasks and create open-source hardware platforms for reproducible experimentation.

## 6 Conclusion

Imitation learning is maturing from a convenience tool into a robust, multimodal paradigm for agile legged robots. Our taxonomic analysis reveals several critical insights about the current state of imitation learning for legged robots. Behavior cloning remains the dominant approach, appearing in almost half of the surveyed works, demonstrating its practical effectiveness and hardware-friendly implementation characteristics. However, the field is experiencing rapid diversification, with adversarial motion priors, diffusion-based methods, and emerging techniques such as decaying action priors and MPC distillation gaining significant traction.

The data landscape has undergone a fundamental shift, with model-predictive control logs now representing the most frequently used training data source, surpassing traditional animal and human motion capture approaches. This transition reflects the field’s growing emphasis on morphologically consistent, scalable data generation methods that can produce noise-free demonstrations while maintaining physical feasibility. The convergence of several technological trends suggests promising directions for the field. LLM-based generative models are beginning to enable semantic control of locomotion behaviors, while torque-based control approaches offer enhanced compliance and dynamic responsiveness.

Imitation learning has attempted to address one of robotics’ fundamental challenges: eliminating the need for hand-engineered reward functions while achieving natural, efficient locomotion behaviors. By enabling robots to learn directly from demonstrations—whether from animals, humans, or optimized controllers—this paradigm has accelerated development cycles, reduced hyperparameter sensitivity, and provided natural scalability pathways for complex behaviors.
